# Rapidly modifiable factors associated with full vaccination status among children in Niamey, Niger: A cross-sectional, random cluster household survey

**DOI:** 10.1371/journal.pone.0249026

**Published:** 2021-03-31

**Authors:** Mika Kondo Kunieda, Mahamane Laouali Manzo, Akira Shibanuma, Masamine Jimba

**Affiliations:** 1 Department of Community and Global Health, Graduate School of Medicine, The University of Tokyo, Tokyo, Japan; 2 Department of Global Health and Population, Takemi Program in International Health, Harvard T.H. Chan School of Public Health, Boston, Massachusetts, United States of America; 3 Ministry of Public Health, Niamey, Niger; Iwate Medical University, JAPAN

## Abstract

**Background and objectives:**

Vaccination status becomes more equitable when interventions are carried out to eliminate poverty or to improve levels in maternal education. Low-income countries need to identify interventions that would have a more immediate and equitable effect. The present study aimed to identify rapidly modifiable factors associated with full vaccination status among children in Niamey, Niger.

**Methods:**

A cross-sectional, random cluster household survey was conducted in Niamey’s five health districts. Data on vaccination coverage and socioeconomic household characteristics were collected. Logistic regression analysis was conducted with data on 445 mothers and their children aged 12–23 months.

**Results:**

Of 445 children, 38% were fully vaccinated. Mothers who were satisfied with their health worker’s attitude and had correct vaccination calendar knowledge (adjusted odds ratio [aOR] 5.32, 95% confidence interval [CI] 2.05–13.82) were more likely to have fully vaccinated children. Mothers who had completed secondary school (aOR 2.04, 95% CI 1.17–3.55) were also associated with having fully vaccinated children.

**Conclusions:**

A higher rate of full vaccination among children could be achieved by relatively short-term modifiable factors. These modifiable factors are mothers’ satisfaction with health workers’ attitudes and knowledge of the vaccination calendar. Maternal satisfaction with health workers’ attitudes could be improved through better interpersonal communication between health workers and mothers. Specifically, mothers should be given specific information on time intervals between appointments. Strengthened communication interventions may be effective in improving both the acceptability of health services and low vaccination coverage.

## Introduction

In Niger, there is a relatively high rate of initial access to vaccination. For example, results from the 2012 Demographic Health Surveys (DHS) showed coverage rates of 84.0% for Bacillus Calmette–Guérin (BCG) and 86.2% for the Diphtheria, Tetanus, and Pertussis vaccines (DTP) 1 among children aged 12–23 months [1]. For DTP 2, the coverage fell to 78.9%. For DTP 3, the coverage fell by another 10%. Only 68.7% of children receive the measles vaccination before they turned 1-year-old. Of the 2275 children surveyed in 2012, the full vaccination coverage was estimated at 52.0%, with 4.1% of children having never received any vaccination.

Globally, vaccination coverage is monitored through DTP coverage. In Niger, the pentavalent vaccine (Penta) coverage is used as Penta is a combination of five vaccines: those for DTP, hepatitis B, and *Haemophilus influenza* type b. A Penta coverage ≥80% implies good access to services [2]. A poor utilization rate reflects a high drop-out rate ≥10% [3]. Niger has good accessibility but low utilization, a trend that has continued even after user fees were exempted for maternal and child services [4]. As accessibility and availability of services are not problematic, acceptability becomes the issue. In the field, officials frequently blame the high vaccination drop-out rate on socioeconomic factors, notably the lack of maternal education.

Vaccination status becomes more equitable when interventions are carried out to eliminate poverty or to improve maternal education levels. However, such interventions require time to take effect. For example, years of maternal education or maternal knowledge can be changed. However, change might take longer than a few years. In addition, there is no assurance that knowledge would lead to behavior change. Poverty elimination in order to achieve equitable vaccination would take decades. As low-income countries (including Niger) have low human capital and limited resources, they cannot afford to wait. These countries need to identify rapid or short-term interventions that would have a more immediate and equitable effect in a year or two.

In this context, the present study aimed to identify rapidly modifiable factors associated with full vaccination status among children in Niamey, Niger.

## Materials and methods

### Study design

This cross-sectional study was conducted in October 2016. A multi-stage random cluster design was used, following the WHO cluster survey 2005 guidelines [5]. Just as the study was being implemented, the WHO cluster survey 2015 draft working guidelines were published [6].

### Study setting

The study setting is the urban capital of the Republic of Niger, Niamey. A random selection of clusters from the 2012 census and other survey preparations was performed at the National Institute of Statistics (INS) during the first week of October 2016. The questionnaire was pretested at the surveyor training day on October 9. Data were collected through household interviews from October 10 to 14.

### Study participants

Mothers of children aged 12–23 months were identified within each cluster. The inclusion criteria for this study were 1) a parent of children aged 12–23 months at the time of the survey; 2) the child had slept in Niamey the night before the interviewer’s visit; and 3) one child, in the case of multiple children between 12 and 23 months, only data of the youngest child were collected. The exclusion criteria were 1) persons who did not give their consent to be interviewed.

### Study variables

The dependent variable was a fully vaccinated status verified by the child’s vaccination record in the MCH handbook. The present study did not collect information on vaccination based on the mother’s memory.

For the dependent variable of a fully vaccinated child, independent variables derived from the literature were selected. Specifically, independent socioeconomic variables were mostly structural and non-modifiable factors including maternal education level [7–11], socioeconomic status based on wealth quintile [7–10,12], maternal and paternal ages (as continuous variables), maternal employment status [13], and the child’s birth order [14,15]. Independent and modifiable variables included in the analysis were: discussion about vaccination with the family [16–19], satisfaction with the attitudes of health workers [13,18,20–22], and maternal knowledge of the vaccination calendar. All independent variables were binary.

Vaccination status was encoded as “1” if the child had received BCG, three DTP doses, oral polio vaccine drops thrice, and one dose of measles vaccine. If the child had missed any of the vaccines mentioned above, the antigen status was encoded as “0”. Full vaccination status was the sum “8” for 8 vaccines which were coded “1”. If all antigens were coded “0”, the child was considered to be “zero-dose” or “not vaccinated”.

The wealth quintiles used in the present study were constructed using principal component analysis of household assets. Questions were asked on electricity, water source, home and vehicle ownership, livestock ownership, as well as ownership of a television set, refrigerator, and air conditioner. Typical DHS questions regarding housing materials were not included, as roofing and flooring materials used in Niamey are similar.

Binary response questions were asked on satisfaction with the attitudes of health workers, and whether the women discussed vaccination with friends and family. Responses were coded in binary form (“1” for “yes” and “0” for “no”).

Maternal knowledge of the vaccination calendar was assessed through the question, “How many times should you take your child (to the vaccination center) so that he/she is fully vaccinated?” Mothers who correctly replied “five” to the question, received a code of “1” for “has knowledge”. Mothers who gave another number or were unable to answer received a code of “0” for “does not have knowledge”.

### Data sources and measurement

The sample size for the five stratified health districts was calculated based on an 85% likely coverage for DTP 3 from administrative data obtained in 2014 (8). In addition, the following parameters were applied: a 95% confidence interval (CI), 90% power, and an intra-cluster correlation of 0.042 (or a design effect of 1.38, which indicated very little variation in coverage). After stratifying according to health district, 46 clusters were selected randomly from a list of enumeration areas (EAs). This cluster list was constructed for the 2012 Census by the INS of Niger.

Study questions were drafted based on the 2012 Niger DHS questionnaire and WHO cluster survey 2015 draft working guidelines [1,6]. A small study coordination committee reviewed the questions for cultural appropriateness. The questionnaire (only available in French) has been uploaded to Harvard Dataverse. The study coordination committee then pretested the questions while training 22 surveyors. Four of the 22 were selected as supervisors during training to oversee the remaining 18 surveyors. The four supervisors were supervised by the study coordination committee.

The first household to be assessed in the study was marked randomly on maps prepared by INS. Cluster boundaries were confirmed on the ground. Surveyors then canvassed each cluster, going in a predetermined direction. The surveyors continued until they had interviewed ten mothers. The same procedure was repeated in each of the 46 clusters. For clusters that were markets or bus terminals, surveyors randomly approached mothers carrying babies on their backs. Mothers were interviewed using a structured questionnaire.

Missing data on vaccination coverage were supplemented with information from a photo of the vaccination page of the person’s MCH handbook. Other missing information on the husband’s education level and other variables were not supplemented or imputed.

### Data analysis

Mother–child pairs were nested within their neighborhoods. Usually, when data are nested, a multilevel regression is run. However, the intraclass correlation coefficient (0.02, standard error 0.03) was negligible when an empty model was run for the dependent variable of a fully vaccinated child. This was interpreted as minimal variation in the dependent variable from one neighborhood to another. Therefore, data were analyzed at the mother–child level without considering neighborhood clustering. A single-level logistic regression analysis was run. The data were checked for multicollinearity. All statistical analyses were performed using Stata version 13.1 (StataCorp LLC, College Station, TX, USA). The full dataset has been uploaded onto Harvard Dataverse.

### Ethics approval

The Research Ethics Committee of the Graduate School of Medicine, University of Tokyo approved the protocol of the present study (serial number 11218). The National Ethics Committee of Niger authorized study execution (no. 03660/MSP/SG/DEP/DER). Consent in the form of a signature or thumbprint was obtained from all mothers. The Research Ethics Committee waived the need to obtain parental consent as married Nigerien women (even if they are teenagers) are not considered minors. Participation was strictly voluntary, with no incentive given. Measures were taken to ensure the confidentiality of the data so that individuals could not be identified.

## Results

### Study population characteristics

A total of 460 mothers of children aged 12–23 months were recruited. Data regarding 15 children were removed before data analysis, as they did not meet the age inclusion criteria.

Table 1 shows the descriptive characteristics of the 445 mothers. Of 146 mothers who were unable to read and write, 101 (69.2%) did not have fully vaccinated children. More than half (n = 96, 52.8%) of 182 mothers who had completed both primary and secondary schools did not have a fully vaccinated child. Of 445 mothers, 436 were divided into quintiles according to their socioeconomic status. The poorest quintile had the lowest percentage (23.5%), while the middle class had the highest percentage (49.4%) of fully vaccinated children. The richest quintile had only 38 fully vaccinated children out of 88 (43.2%), less than that of the middle class.

**Table 1:** Study population characteristics.

Variable	Total		Child not fully vaccinated		Child fully vaccinated		p-value
	n	(%)	n	(%)	n	(%)	
**Mother’s education level (n = 445)**							0.030
Unable to read and write	146	(32.8)	101	(69.2)	45	(30.8)	
Able to read and write	28	(6.3)	19	(67.9)	9	(32.1)	
Primary and secondary	182	(40.9)	96	(52.8)	86	(47.3)	
Post-secondary	26	(5.8)	16	(61.5)	10	(38.5)	
Koranic	63	(14.2)	42	(66.7)	21	(33.3)	
**Socio-economic status (measured by constructed wealth index) (n = 436)**							0.003
Poorest	85	(19.5)	65	(76.5)	20	(23.5)	
Poorer	89	(20.4)	60	(67.4)	29	(32.6)	
Middle	87	(20.0)	44	(50.6)	43	(49.4)	
Richer	87	(20.0)	48	(55.2)	39	(44.8)	
Richest	88	(20.2)	50	(56.8)	38	(43.2)	
**Mother’s age group (n = 443)**							0.721
15–19 years	32	(7.2)	19	(59.4)	13	(40.6)	
20–24 years	103	(23.3)	63	(61.2)	40	(38.8)	
25–29 years	132	(30.0)	86	(65.2)	46	(34.9)	
30–34 years	80	(18.1)	50	(62.5)	30	(37.5)	
35–39 years	73	(16.5)	43	(58.9)	30	(41.1)	
≥40 years	23	(5.2)	11	(47.8)	12	(52.2)	
**Father’s age group (n = 437)**							0.038
20–29 years	44	(10.1)	28	(63.6)	16	(36.4)	
30–34 years	84	(19.2)	46	(54.8)	38	(45.2)	
35–39 years	96	(22.0)	69	(71.9)	27	(28.1)	
40–44 years	91	(20.8)	60	(65.9)	31	(34.2)	
45–49 years	69	(15.8)	40	(58.0)	29	(42.0)	
≥50 years	53	(12.1)	25	(47.2)	28	(52.8)	
**Mother’s employment status (n = 445)**							0.651
Stay-at-home mother	341	(76.6)	208	(61.0)	133	(39.0)	
Working mother	104	(23.4)	66	(63.5)	38	(36.5)	
**Child’s birth order (n = 444)**							0.689
1^st^ child	92	(20.7)	53	(57.6)	39	(42.4)	
2^nd^ child	84	(18.9)	52	(61.9)	32	(38.1)	
3^rd^ child	87	(19.6)	49	(56.3)	38	(43.7)	
4^th^ child	55	(12.4)	37	(67.3)	18	(32.7)	
5^th^–6^th^ child	73	(16.4)	47	(64.4)	26	(35.6)	
7^th^–11^th^ child	53	(11.9)	35	(66.0)	18	(34.0)	

(n = 445 total, n = 274 not fully vaccinated, n = 171 fully vaccinated).

Children in the poorest quintile compared to those in the richest quintile were disadvantaged by 19.7% in terms of full vaccination status. Children whose mothers were uneducated were disadvantaged by 16.5% in terms of full vaccination status, compared to those whose mothers received secondary education. A pattern of economic- and education-related inequality similar to that reported by the WHO was observed.

One-third of the mothers were aged 25–29 years. Almost one in four mothers were aged between 20–24 years. Three out of four mothers were stay-at-home mothers, and one-fourth replied that they were working mothers. No association was found between maternal employment status and full child vaccination status. One-fifth of the children were first-borns. Two-fifths of the children were second- and third-borns. Of 444 children, 53 (11.9%) were seventh to eleventh-borns. No association was found between a child’s birth order and full vaccination status.

Table 2 shows modifiable factors assessed among the 445 mothers. When mothers were asked “Are you satisfied with the attitude of your health worker?”, 384 of 433 mothers (88.7%) responded positively. Of 433 mothers who were asked the question regarding maternal knowledge of the vaccination calendar, 208 (48.0%) responded correctly. Of these 208 mothers, 101 (48.6%) had fully vaccinated children.

**Table 2:** Study population behavioral characteristics.

Variable	Total		Child not fully vaccinated		Child fully vaccinated		p-value
	n	(%)	n	(%)	n	(%)	
**Mother’s possession of maternal and child health handbook (n = 445)**							0.001
No	18	(4.0)	18	(100.0)	0	(0.0)	
Yes	427	(96.0)	256	(60.0)	171	(40.0)	
**Mother’s discussion with family about vaccination (n = 434)**							0.019
No	119	(27.4)	83	(69.8)	36	(30.3)	
Yes	315	(72.6)	181	(57.5)	134	(42.5)	
**Mother’s satisfaction with health worker’s attitude (n = 433)**							0.025
No	49	(11.3)	37	(75.5)	12	(24.5)	
Yes	384	(88.7)	226	(58.9)	158	(41.2)	
**Maternal knowledge of vaccination calendar (n = 433)**							<0.001
No	225	(52.0)	156	(69.3)	69	(30.7)	
Yes	208	(48.0)	107	(51.4)	101	(48.6)	

(n = 445 total, n = 274 not fully vaccinated, n = 171 fully vaccinated).

### Vaccination coverage

**Fig 1:**
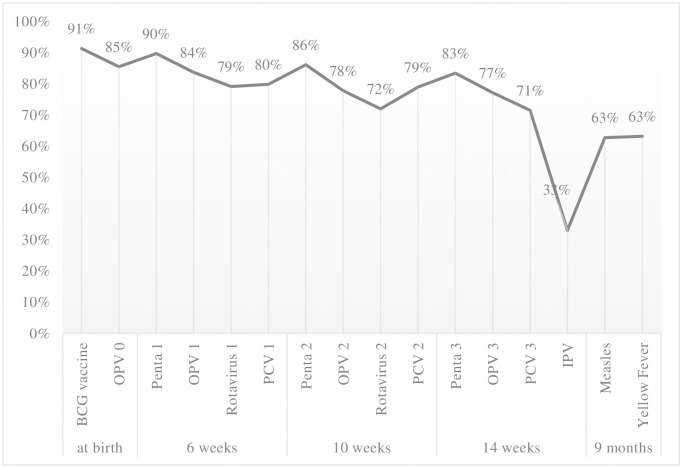
Routine vaccination coverage rates along the vaccination schedule. OPV: oral poliovirus vaccine. IPV: injectable poliovirus vaccine. Penta: Pentavalent vaccine (a 5-in-1 vaccine) containing five antigens (diphtheria, pertussis, tetanus, hepatitis B, and *Haemophilus influenzae* type b). PCV: pneumococcal vaccine.

One-hundred-and-seventy-one (38.4%) of 445 children were fully vaccinated. As shown in Fig 1, 91% of children received the BCG vaccine soon after birth. DTP 1 (Penta 1) is administered when a child is 6 weeks old. The coverage of DTP 1 vaccination was 90%. The DTP 2 (Penta 2) vaccine is given when a child is 10 weeks old. The coverage of DTP 2 vaccination was 86%. The DTP 3 (Penta 3) vaccine is administered when a child is 14 weeks old; the coverage was 83%. The measles vaccine is administered when a child is 9 months old, or 6 months after DTP 3. There was a 20% drop in coverage between DTP 3 and measles. The measles vaccine coverage was 63%.

### Socioeconomic and modifiable factors associated with a fully vaccinated status

Table 3 shows the result of multivariable logistic regression on the association between the independent variables and the dependent variable of a fully vaccinated status. Given the relatively small sample size of the present study, selected socioeconomic variables were included in the logistic regression analysis [23,24]. Mothers in the middle wealth quintile (adjusted odds ratio [aOR] 4.05, 95% CI 1.90–8.66) and the richer wealth quintile (aOR 2.67, 95% CI 1.28–5.58) were likelier to have fully vaccinated children. Mothers who had completed primary and secondary schools (aOR 2.04, 95% CI 1.17–3.55) were likelier to have fully vaccinated children. Mothers who were satisfied with their health workers’ attitudes and had correct knowledge of the vaccination calendar (aOR 5.32, 95% CI 2.05–13.82) were also likelier to have fully vaccinated children.

**Table 3:** Factors associated with full vaccination status^1^ (n = 412).

Factors	aOR	95% CI	p-value
**Mother’s age**	1.04	0.99–1.10	0.129
**Father’s age**	1.03	0.99–1.06	0.132
**Child’s birth order**	0.90	0.77–1.05	0.183
**Maternal education level**			
Unable to read and write (ref)			
Able to read and write	0.80	0.31–2.07	0.650
Primary and secondary	2.04*	1.17–3.55	0.011
Post-secondary	1.35	0.48–3.76	0.570
Koranic	1.28	0.62–2.67	0.502
**Mother’s employment status**			
Stay-at-home mother (ref)			
Working mother	0.58	0.33–1.02	0.059
**Socioeconomic status (measured by constructed wealth index)**			
Poorest (ref)			
Poorer	1.47	0.71–3.05	0.301
Middle	4.05***	1.90–8.66	<0.001
Richer	2.67**	1.28–5.58	0.009
Richest	1.96	0.91–4.20	0.084
**Mother’s discussion with family about vaccination**			
No (ref)			
Yes	1.48	0.88–2.50	0.140
**Mother’s satisfaction with health worker’s attitude and knowledge about vaccination calendar**			
Not satisfied and incorrect knowledge (ref)			
Not satisfied but correct knowledge	2.87	0.67–12.23	0.155
Satisfied but incorrect knowledge	2.26	0.88–5.81	0.092
Satisfied and correct knowledge	5.32**	2.05–13.82	0.001

*: Significant at the 5% level,

**: Significant at the 1% level,

***: Significant at the 0.1% level.

^1^ A child is fully vaccinated when he/she has received one dose of BCG vaccine at birth, three doses of polio and DTP vaccines at six, 10, and 14 weeks after birth, and one dose of measles vaccine at nine months.

## Discussion

The present study identified modifiable and less modifiable factors that were associated with the full vaccination of children. The modifiable factors were mothers’ satisfaction with their health workers’ attitudes and their knowledge of the vaccination calendar. Financial status and education level were considered as less modifiable factors.

### Maternal knowledge of the vaccination calendar and satisfaction with health worker’s attitude

In the present study, mothers who knew that they needed to bring their children to the health center five times for vaccination were more likely to have fully vaccinated children. Maternal education and health literacy levels are commonly associated with higher vaccination coverage [7–11]. However, health literacy level assessment can be not only complicated but also inconsistent [25]. In the present study, maternal knowledge of the vaccination calendar was assessed through a simple and direct question with a binary response. Such a question can easily be added to household and vaccination coverage surveys. In this way, maternal knowledge of the vaccination calendar could predict whether the mother will bring back her child for further vaccination. If the knowledge is insufficient, communication interventions could be implemented to modify this independent variable rapidly. Communication could improve the correct knowledge of the vaccination schedule, such as the time intervals between appointments. Mothers might be nudged to come for timely vaccination.

Children were more likely to be fully vaccinated when their mothers had the knowledge and were satisfied with health worker attitudes. Health worker attitudes and knowledge may be interconnected. Health worker attitudes are critical when a mother is looking for information on vaccination [26–28]. If health worker attitudes are acceptable, mothers are more likely to trust vaccination programs and vaccines. Trusting mothers may be more willing to attend vaccination sessions. Through these visits, mothers might acquire correct knowledge of the vaccination schedule compared to those women who come less often.

### Socioeconomic status and healthcare utilization

In the present study, the poorest and poorer mothers were less likely to have fully vaccinated children. Compared to children in the richest quintile, children in the poorest quintile were disadvantaged, as were children whose mothers were uneducated compared to those whose mothers received secondary education. A pattern of economic- and education-related inequality similar to that reported by the WHO [29] was observed. Similar findings have been published on the association between lower socioeconomic status and the lower likelihood of a fully vaccinated child [8,12,30,31]. One explanation was that poorer mothers had less time or “multiple livelihood activities that deterred clinic attendance” [32]. A previous study also found that poor mothers felt that they were treated poorly by health workers [32]. Health worker attitudes may influence healthcare utilization more than previously acknowledged. Therefore, modifying health worker attitudes may be vital to improving healthcare utilization among poor mothers. More economically disadvantaged children could be fully vaccinated if sympathetic health workers communicated with mothers better. These factors promote vaccination service utilization.

Economically underprivileged children could also be protected from vaccine-preventable infectious diseases through reinforced herd or community immunity. Community immunity can be raised if a critical mass has been vaccinated through routine vaccination and vaccination campaigns [33]. In addition, community immunity can be kicked-off with correct maternal knowledge of the vaccination calendar and helpful health workers, both of which are short-term modifiable factors.

### Study limitations

The present cross-sectional study did not record vaccination antigens received by children of mothers who did not possess their child’s vaccination record or a maternal child health handbook. Recall bias, a problem flagged by prominent researchers in this field [34,35], was therefore minimized. Selection bias is possible as the surveyors may have selected mothers who were able to show their child’s vaccination record. The random cluster survey design prevented selection bias based on neighborhood or neighborhood health centers. Chance responses to the question regarding knowledge of the maternal vaccination calendar could not be controlled. In any case, a binary response question was the most direct way to assess maternal knowledge of the vaccination calendar. The wealth index quintiles need to be interpreted with caution. The wealth quintiles used in the present study were constructed from urban household assets unweighted to a national scale. The results could be skewed, as this study’s urban residents fall in the top two quintiles of the national wealth index. Owing to the cross-sectional design, the cause–effect relationship between modifiable factors and full vaccination status could not be assessed. For example, mothers could have acquired knowledge regarding the vaccination calendar while getting their child fully vaccinated or mothers could have learned the correct vaccination calendar through frequent contact with health workers. Finally, an ethnographic and spatial analysis could have furthered understanding of urban specificities, including place and access to health services.

## Conclusions

Based on proof of vaccination recorded in the MCH handbook, only 38% of children were fully vaccinated. A higher rate of full vaccination among children could be achieved by relatively short-term modifiable factors. These modifiable factors are mothers’ satisfaction with health workers’ attitudes and knowledge of the vaccination calendar. Maternal satisfaction with health workers’ attitudes could be improved through better interpersonal communication between health workers and mothers. Specifically, mothers should be given specific information on time intervals between appointments. Strengthened communication interventions may be effective in improving both the acceptability of health services and low vaccination coverage. To increase the number of fully vaccinated children, the coverage of the last vaccine (measles vaccine) needs to be improved.
